# DATS_2022: A versatile indian dataset for object detection in unstructured traffic conditions

**DOI:** 10.1016/j.dib.2022.108470

**Published:** 2022-07-14

**Authors:** Bhakti A Paranjape, Apurva A Naik

**Affiliations:** MIT World Peace University, Pune, Maharashtra, India

**Keywords:** Machine Learning, Annotation, Pre-trained Models, Localization

## Abstract

Driver Assistance System has proved to be the best alternative to the autonomous vehicles in countries like India which has heavy and irregular traffic conditions. It is a challenging task for a machine to visually understand the traffic scenes in a complex environment. To develop a machine learning algorithm for such an environment, there is a need of a dataset that may provide the correct information of the traffic scene. The proposed dataset, DATS_2022, is a comprehensive dataset for object detection specific to Indian traffic scenario. The dataset consists of images captured using a high-resolution camera in an android phone. The images are annotated using a free, open-source tool. XML files for these annotations are generated and saved which are used to extract the labels for training various machine learning algorithms. DATS_2022 is a complete dataset with images from rural as well as urban Indian traffic scenes. The dataset consists of more than 10000 images with 45object classes. There are more than 7000 annotations in different formats along with the dataset till the submission of this work. It aims to support research in the area of object detection and classification using deep and machine learning algorithms.

## Specifications Table

**Table:** 

Subject	Computer Vision and Pattern Recognition, Computer Science Applications, Artificial Intelligence
Specific subject area	A dataset to provide an improved and annotated training data that would build a high-quality machine learning model for object detection
Type of data	Image and Annotations
How the data were acquired	The images in the dataset were captured using a high-resolution android phone (Redmi Note 8 Pro and Redmi Note 5 Pro) that has 64 MP super camera. A mix of photo and video mode of the camera was used to capture the images. Video sequences, 10-15 sec long with 30 frames per second (FPS) were recorded on various locations at different times of the day. VLC Media Player was used to extract the frames from the video sequences.
Data format	Raw images in .jpeg and .png format. Annotation files in .xml, .txt and .json format
Description of data collection	DATS_2022 is generated by the author alone as a part of her research work. The author collected the images while travelling on a 2-wheeler bike or a car in different parts of the residing city as well as other cities and villages as mentioned in the later section. The dataset contains images captured using a personal high-resolution android phone (Redmi Note 8 Pro and Redmi Note 5 Pro) that has a 64 MP super camera, and no professional photographer was involved in the process. The images were captured during different times of the day and during all seasons of the year. The weather during these seasons was different and as the location changed the local climatic conditions also changed. The images from rural roads, highways with construction work in progress, roads with dense traffic, roads in the hilly regions, roads with traffic during daytime, evening time and night time, highways with heavy vehicles, roads with animals, etc. are a part of this dataset.
Data source location	Images are collected from different locations in the state of Maharashtra, India
Data accessibility	Repository name: Mendeley Data DATS_2022 Version 2 Published:10 May 2022 DOI:10.17632/nfc34n8svj.2https://data.mendeley.com/datasets/nfc34n8svj/2

## Value of the Data

• The dataset includes images from unstructured traffic environments specific to Indian roads. The standard available datasets contain the images from well-structured traffic scenes from different parts of the world. The Vehicle classes like bikes and rickshaws, animals and animal driven carts are not found in the standard datasets from around the world and are unique to Indian road scenario.
• Very few datasets are available exclusively for Indian roads and hence DATS_2022 is important for the researchers in India. The available datasets [1], [2], [4], [5], [6] are specifically generated for the autonomous vehicles whereas DATS_2022 can be used for any general application.
• Most of the available datasets are not fully annotated and hence cannot be used directly for training the machine learning models. DATS_2022 includes the images as well as corresponding annotations in .xml (pascal VOC), .txt (YOLO) and. json (Create ML) formats.
• Most of the available datasets contain redundant images as they are the images extracted from video sequences. In DATS_2022 every 7^th^ image is extracted to reduce redundancy. The images are captured in photo mode hence repetition of images is avoided.
• All the images were captured by the author alone, using a normal personal android phone camera, over a period of 1 year and there is no team or organization involved. As a result, the number of images is less compared to other datasets. The collection of images would be an ongoing process and the dataset would be updated from time to time.
• The dataset could be used by the research community working in the fields of Autonomous Vehicles and Driver Assistance System, 5G technology which supports the Vehicle to everything (V2X) communication in which each vehicle is connected to every other entity, Internet of Things, Intelligent Transportation systems, Embedded Software for Automotive applications, etc. Google Maps in navigation systems can use the dataset so that it can provide the information regarding the objects present on the road ahead along with the route and hence the common people using all the above technologies or applications would be largely benefited. This also highlights the social impact of the work.
• The researchers who desire to use this dataset can directly download images from the Mendeley data, https://data.mendeley.com/datasets/nfc34n8svj/2 or can directly contact the author.

## 1 Data Description

DATS_2022 consists of more than 10000 images captured using a high-resolution android phone (Redmi Note 8 Pro and Redmi Note 5 Pro) that has a 64 MP super camera. The picture quality of these cameras is comparable with the commercial cameras. In recent times increased use of mobiles is seen and is preferred over bulky cameras. So, the images captured using a mobile camera would make a more practical or realistic dataset.

The images were captured in various parts of the state of Maharashtra, India. The different locations, type of roads and the traffic conditions are given in Table 1.

**Table 1:** The different locations where images are captured and the traffic conditions

Location	Type of Roads	Structured / Unstructured
In and around Pune (second-largest city in the state of Maharashtra, India)	Urban as well as rural roads	Mix of structured and unstructured environment

Pune Bangalore National highway (NH 48) (National Highway of India traversing through seven states of India. It has a total length of 2807 km)	6 lanes in some of the parts	Structured

Mumbai Goa National Highway (NH 66) (a 4 lane 1,608 km long busy National Highway that runs roughly north–south along the western coast of India, parallel to the Western Ghats)	Road under construction with 4 lanes	Structured but stray animals are found in most of the parts

Amboli (a mountain pass in the Sahyadri. It is a hill station in south Maharashtra, India)	Covers the roads in the hilly region with high rising mountains on one side and deep valley on the other	Unstructured

Sawantwadi (a town in Sindhudurg district of Maharashtra, India) and villages around	Covers rural area	Unstructured

Kolhapur (a city in the southern part of the Indian state of Maharashtra, India)	Covers the traffic on highways	Mix of structured and unstructured environment

The images were captured during different times of the day like ‘Day’, ‘Evening’ and ‘Night’ time and during all seasons of the year. The locations cover a wide range from crowded city roads, 4-lane national highways with heavy as well as moderate traffic, roads with animals moving around to lonely roads, etc.

**Fig. 1:**
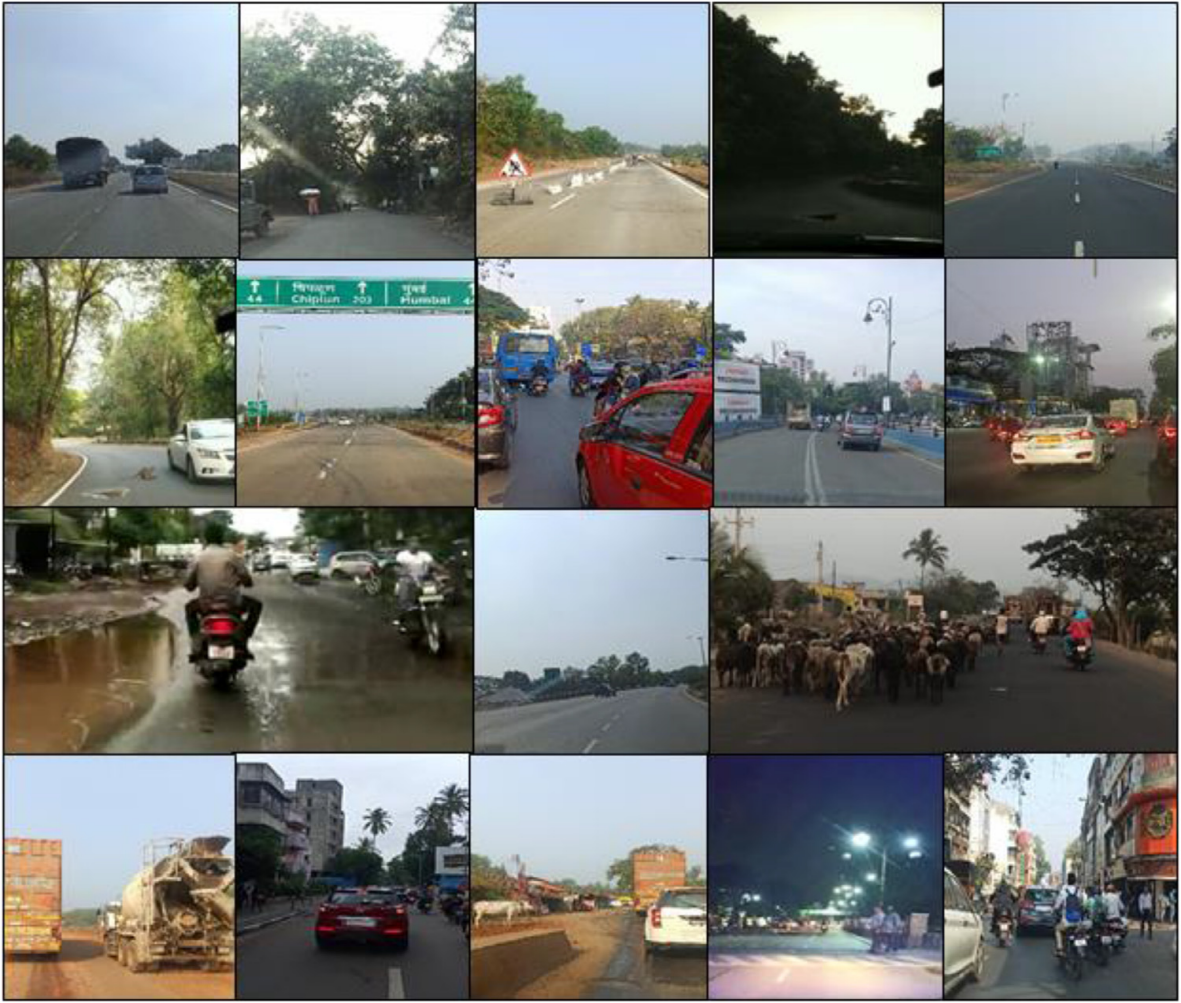
Images of diverse road conditions at different time of the day

Fig. 1 includes the images that show the national highways, rural roads, highways with construction work in progress, crowded city roads, roads in the hilly area with monkeys, traffic during daytime, evening time and night time, highways with heavy vehicles, the existence of animals on roads, etc. The roads in the hilly area are with lots of sharp turns and dense forest on one side or high rising mountains and deep valleys on the other side. The roads through the rural villages are covered with trees on both sides.

There are locations where vehicles are not present but some objects like the road dividers, lamp posts, traffic signals, boards, etc. could be captured. Some of the images include different animals like dogs, goats, cattle, camel, and horse, which is a common scene on Indian roads. ‘Animals’, are not seen in developed countries and hence not considered in any of the standard datasets. The images are captured during various seasons throughout the whole year. The weather during these seasons was different and as the location changed the local climatic conditions changed too. All above aspects together increase the diversity of the dataset.

## 2 Experimental Design, Materials and Methods

**Fig. 2:**
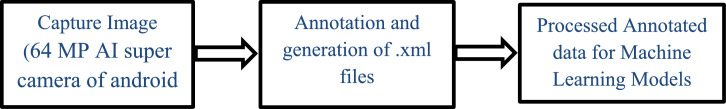
Dataset generation process

Fig. 2 explains the process of dataset generation. The images are captured on all possible roads in the city of Pune and some of the places around Pune, Maharashtra, India. The objective of the dataset was to collect images from different road conditions in various parts of the city and at different weather conditions as well as different times of the Day. The camera of the android mobile phone was used to capture images. High resolution images were captured using this camera. Video sequences were also recorded and frames were extracted to save as image using the VLC media player.

**Fig. 3:**
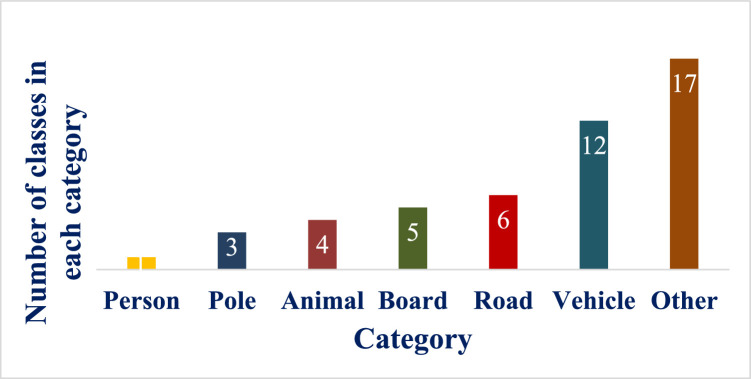
Different Categories and corresponding Classes in each Category

In DATS_2022, objects are classified into 45 different classes. They are represented with broad categories and different classes in each category in Fig. 3.

A good dataset should also provide the data description also called as annotations along with the images. The process of defining regions / objects in an image and creating a textual description of those regions / objects is known as data annotation.

The images in the proposed dataset are annotated using the LabelImg tool [3] which is a free open-source tool. Once all the images are annotated and saved, xml file of each image is generated in the specified folder. The xml file contains the information of each object labeled and the position of that object in that image. It also specifies the type of annotation: ‘bounding box’ in this case. Similarly, .txt and .json files are also generated and saved in specified folder.

**Fig. 4:**
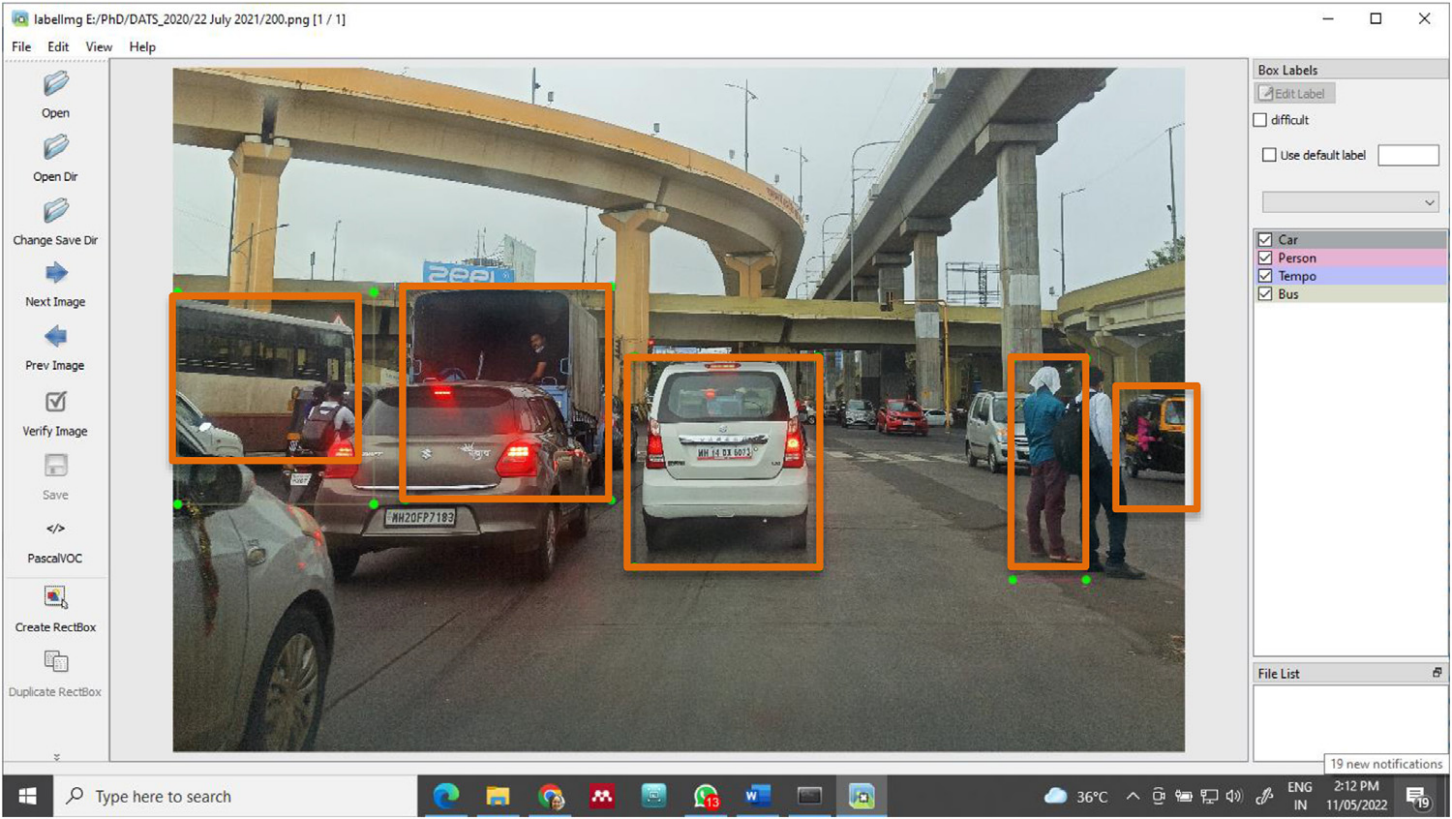
Annotated image using LabelImg Tool

Fig. 4 shows images annotated with different classes using the LabelImg tool using bounding boxes. These images along with the corresponding xml files are ready to be applied to any good machine learning algorithm.

**Fig. 5:**
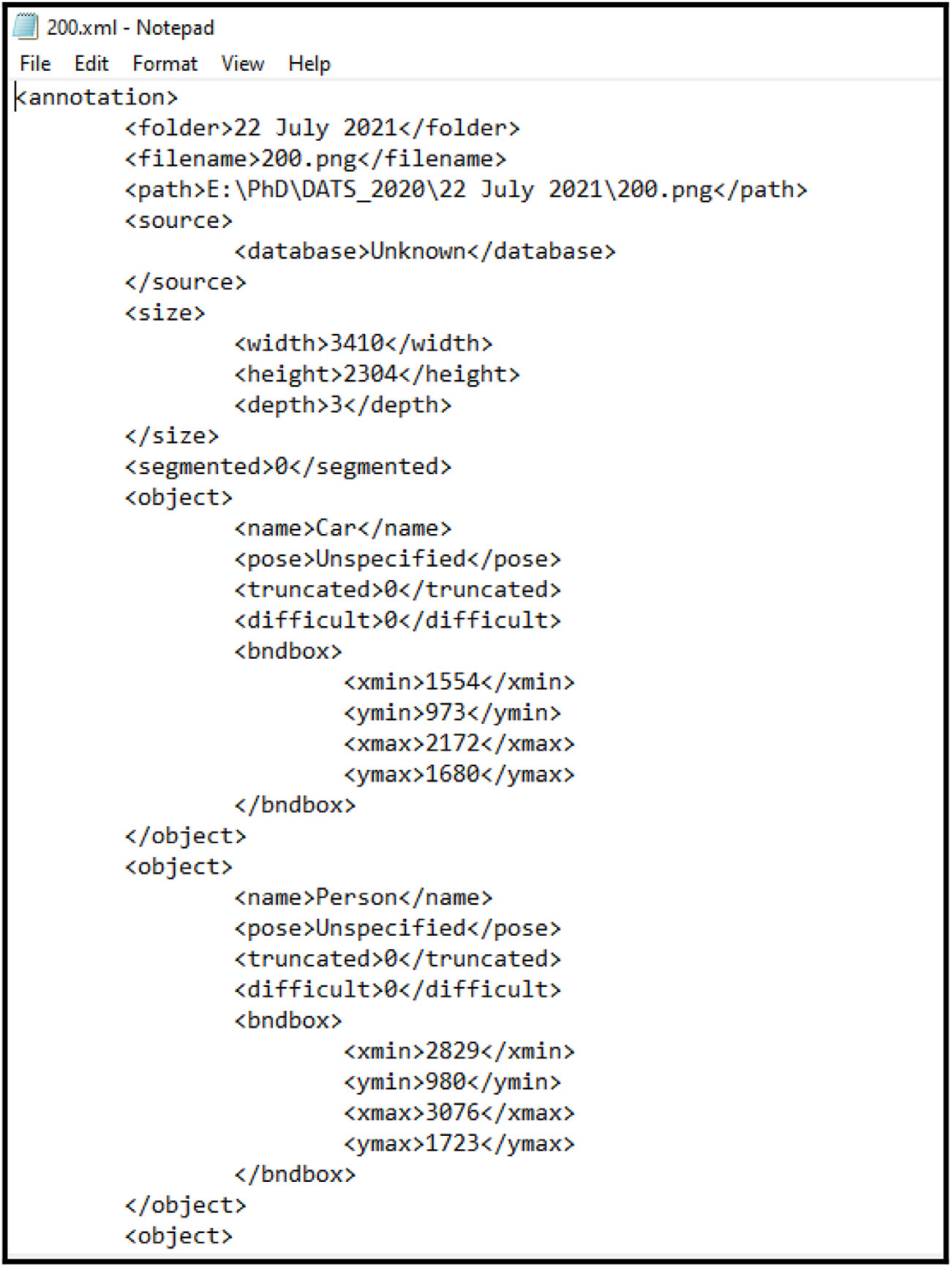
xml file generated after using LabelImg Tool

Fig 5 shows a sample .xml file that is generated after annotating the images using the LabelImg tool and saving in the specified folder.

The focus of this dataset is localization algorithms. Localization algorithms locate the objects in the input image and draw a bounding box around that object specifying its class. DATS_2022 can be readily used for the localization.

The dataset DATS_2022 is published in Mendeley Data [7] as Version 2. It includes 1000 out of the total 10,000 images, since up to 1 GB of data could be uploaded in Mendeley. All 1000 images have corresponding .xml, .txt and .json files included. The researchers involved in the study of developing different algorithms for self-driving cars or driver-assistance systems, can use the images along with the annotations. Having images along with annotations saves a lot of time of the researchers.

## Ethics Statements

The work adheres to Ethics in publishing standard.

## CRediT Author Statement

**Bhakti Paranjape:** Conceptualization, Methodology, Data Curation, Writing – original draft preparation; **Dr. Apurva Naik:** Conceptualization, Supervision.

## Declaration of Competing Interest

The authors declare that they have no known competing financial interests or personal relationships that could have appeared to influence the work reported in this paper.

## Data Availability

DATS_2022 (Original data) (Mendeley Data).
